# Supplementary material for: Integrative Multiomics Analysis of the Heat Stress Response of Enterococcus faecium
Source: Biomolecules. 2023 Feb 25;13(3):437. doi: 10.3390/biom13030437 (PMC10046512; doi:10.3390/biom13030437)
Supplement: Supplementary file 1 [file biomolecules-13-00437-s001.zip › biomolecules-2144734-supplementary.pdf]

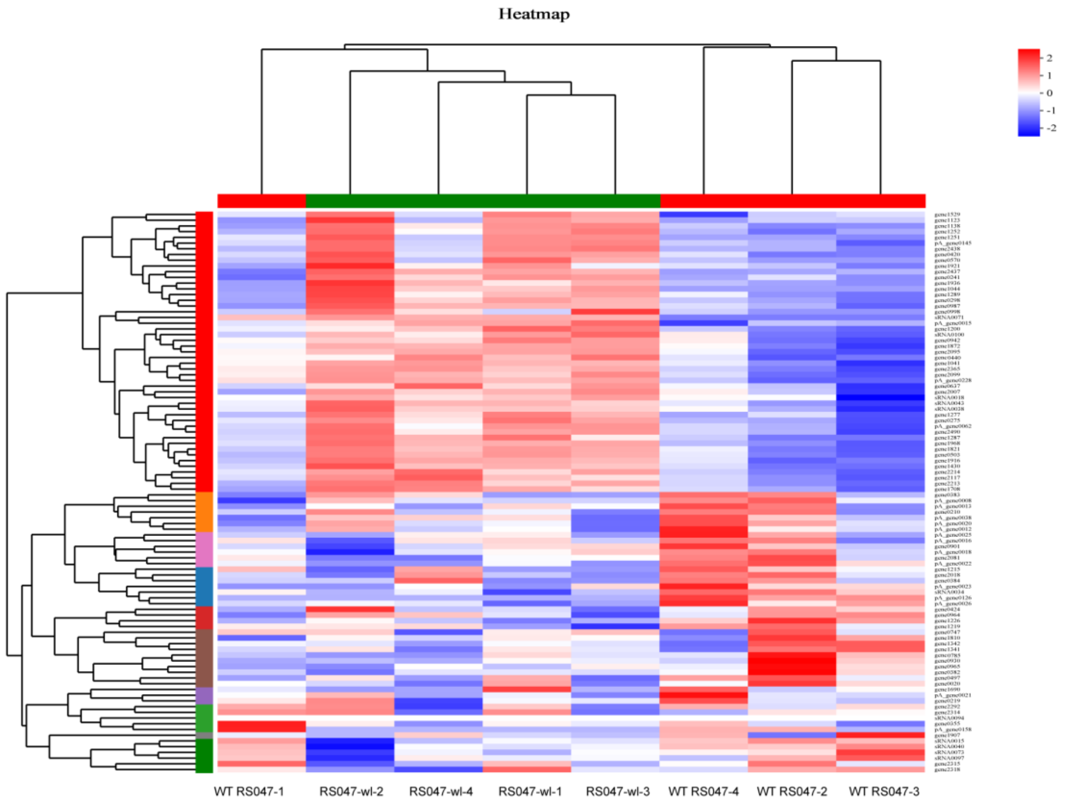

Figure S1. Heat map analysis of 98 DEGs of WT RS047 and RS047-wl.

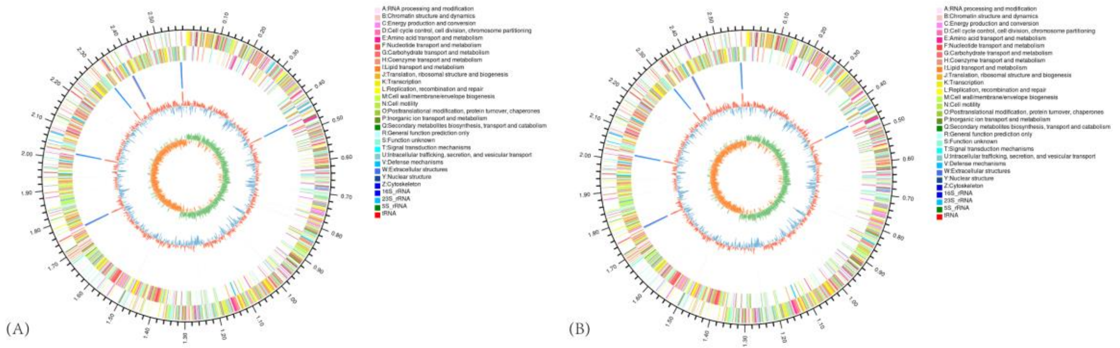

Figure S2. Circos whole-genome map across the WT RS047 and RS047-wl strains, (A) WT RS047, (B) RS047-wl.

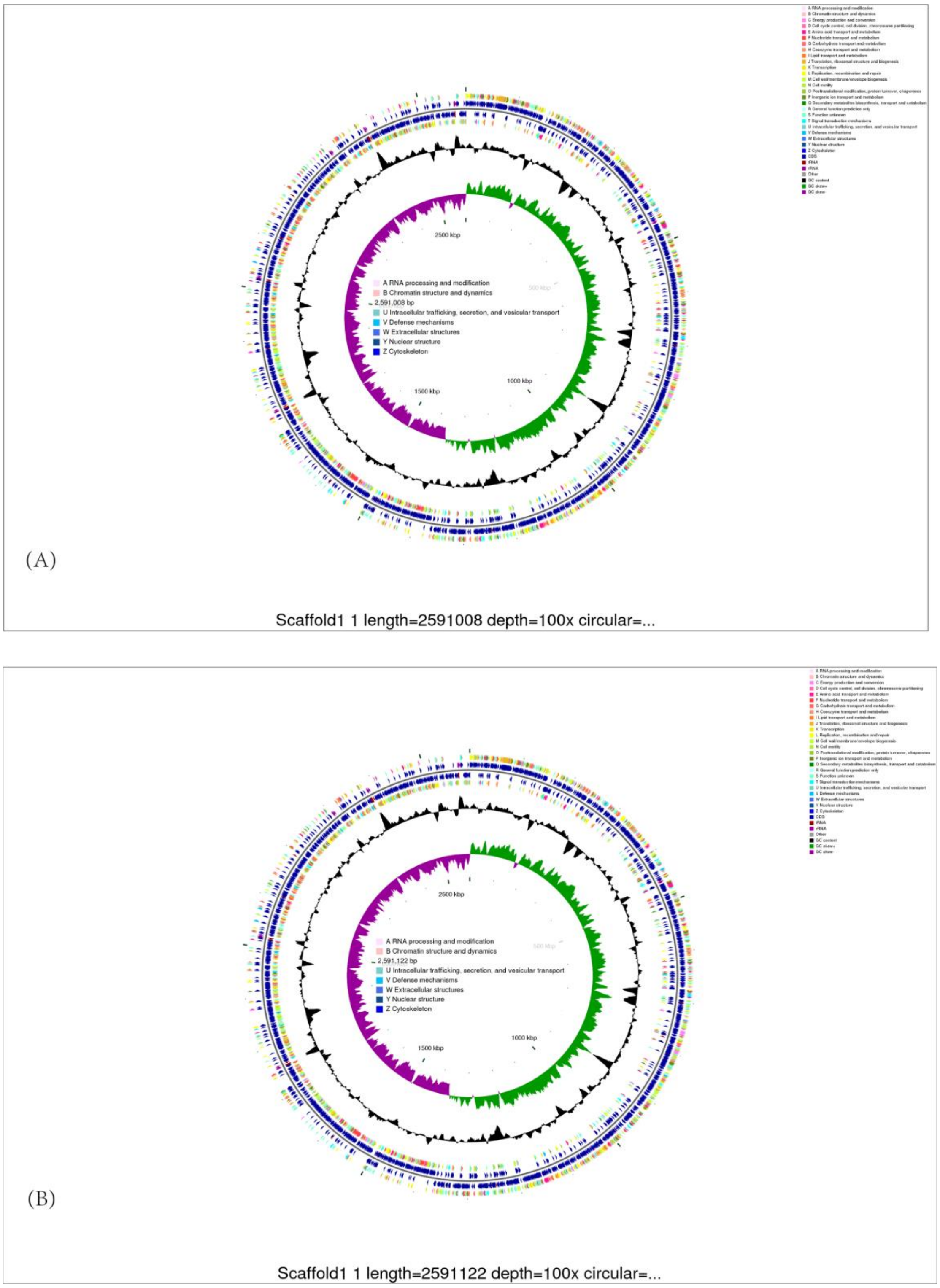

Figure S3. CGView whole-genome map across the WT RS047 and RS047-wl strains, (A) WT RS047, (B) RS047-wl.

Table S1. Amplification primers for candidate differential expression genes

Number	Primer name	Primer sequence (5'→ 3')
1	AgrA	F:TCTCAATGCGGCCAAGATCAGAAC<br>R:ATCGCTACGCCATCCATTTCACTG
2	AgrB	F:GGTTCCTTTATCGCACAAGACCAAC<br>R:CCGATCAGCGTGAGGCTTATTCC
3	AgrC	F:TGCCGGATTGAGACAAGAAGAAGC<br>R:TTAGACCAATCCCACGTTCTTTCCC
4	OpuBD	F:TTATCGGGATGTGGCAAAAGGGATG<br>R:TCGTTGGCATAGCAATAGGCAGTTC
5	uxac	F:AAAGTTCTCCCTTCATTCCGTCCAG<br>R:GCTGCTTCTTCCAGTAGTGTGATCC
6	cd	F:TGGAGAAGACAGCACGGATTGTTTC<br>R:TTCACCCATTCTGGTACAACGAAGC
7	hprT	F:AGCGGTACCATTCATGGCAGATATC<br>R:CCCTGACGAGACTGTTGCATTCC
8	purS	F:CCATCGTATGGGATATGAAACAATCGA<br>R:GCAAGCAACTTGTCACAAATCGT
9	ENT	F:TACTGACAAACCATTCATGATG<br>R：AACTTCGTCACCAACGCGAAC

Table S2. The mutant genes for WT RS047 and RS047-wl

WT RS047						
Gene ID	Gene Name	Gene Description	Location	Start	End	Length (bp)
pA_gene0229	-	IS256 family transposase	PlasmidA	216953	216828	126
gene0267	-	IS982-like element ISEfm1 family transposase	Chromosome	266790	265882	909
pA_gene0077	-	IS3 family transposase	PlasmidA	66832	66188	645
gene1828	-	IS30-like element IS6770 family transposase	Chromosome	1845557	1846426	870
pA_gene0156	-	IS6-like element ISS1D family transposase	PlasmidA	147887	147798	90
pA_gene0127	-	IS6 family transposase	PlasmidA	117661	117503	159
pA_gene0258	-	hypothetical protein	PlasmidA	235109	234969	141
RS047-wl						
Gene ID	Gene Name	Gene Description	Location	Start	End	Length (bp)
pA_gene0231	-	IS256 family transposase	PlasmidA	218888	218544	345
gene1828	-	integrase	Chromosome	1845557	1846426	870
pA_gene0157	-	IS6-like element ISS1D family transposase	PlasmidA	149833	148535	1299
pA_gene0009	-	MULTISPECIES: IS200/IS605 family transposase	PlasmidA	7584	8702	1119
pA_gene0078	-	IS3 family transposase	PlasmidA	67951	66992	960
pA_gene0128	-	IS6 family transposase	PlasmidA	118912	117791	1122
pA_gene0192	-	hypothetical protein	PlasmidA	182429	183277	849

Table S3. Major DEGs of the WT RS047 and RS047-wl that identified by NCBI. Fold change (FC) represents ratio of level in treatment to control. P-values were calculated by univariate analysis (t-test).

Gene_id	Gene name	Gene description	FC(treatment/control)	P-value	Significant	Regulate
gene0998	rimI	MULTISPECIES: ribosomal protein S18-alanine N-acetyltransferase	6.977	0.038696006	yes	up
gene1252	agrA	MULTISPECIES: response regulator transcription factor	4.574	0.008697274	yes	up
gene2214	trmL	tRNA (uridine(34)/cytosine(34)/5-carboxymethylaminomethyluridine(34)-2'-O)-methyltransferase TrmL	3.994	0.000308958	yes	up
gene1529	purS	phosphoribosylformylglycinamidine synthase, purS protein	3.128	0.008810087	yes	up
gene1936	rsmI	16S rRNA (cytidine(1402)-2'-O)-methyltransferase	3.114	0.001563349	yes	up
gene1251	agrC	hypothetical protein	2.942	0.018287178	yes	up
gene1123	aroK	MULTISPECIES: shikimate kinase	2.262	0.024538781	yes	up
gene0987	gluP	MULTISPECIES: rhomboid family intramembrane serine protease	2.261	0.003422923	yes	up
gene1289	ganP	MULTISPECIES: sugar ABC transporter permease	2.253	0.011005642	yes	up
gene1708	araD	L-ribulose-5-phosphate 4-epimerase	2.253	0.04254019	yes	up
gene1968	glnR	MULTISPECIES: MerR family transcriptional regulator	2.147	4.01482E-06	yes	up
gene1430	rnz	MULTISPECIES: ribonuclease Z	2.088	0.000476043	yes	up
gene1287	cd	MULTISPECIES: alpha-glycosidase	2.008	0.00628798	yes	up
gene1872	sfsA	DNA/RNA nuclease SfsA	2.005	0.007502359	yes	up
gene2365	hprT	MULTISPECIES: hypoxanthine phosphoribosyltransferase	2.005	0.010260747	yes	up
gene0440	agrB	MULTISPECIES: accessory gene regulator B family protein	2.003	0.029558535	yes	up
pA_gene0038	topB	DNA topoisomerase 3	0.496	0.033303339	yes	down
gene1690	fruA	MULTISPECIES: PTS sugar transporter subunit IIA	0.487	0.015096454	yes	down
gene0210	opuBD	MULTISPECIES: ABC transporter permease	0.485	3.7746E-05	yes	down
gene0747	trkH	MULTISPECIES: Trk family potassium uptake protein	0.484	0.002072773	yes	down
gene1907	manY	MULTISPECIES: PTS mannose/fructose/sorbose transporter subunit	0.475	0.001121247	yes	down

		IIC				
gene0383	tyrDC	MULTISPECIES: tyrosine decarboxylase	0.469	0.001305088	yes	down
gene0384	gadC	MULTISPECIES: amino acid permease	0.468	0.002338234	yes	down
gene2314	celB	MULTISPECIES: PTS cellobiose transporter subunit IIC	0.467	0.024420165	yes	down
gene2292	uxaC	MULTISPECIES: glucuronate isomerase	0.465	4.19239E-05	yes	down
gene0930	rplS	50S ribosomal protein L19	0.445	0.000898868	yes	down
gene2318	celA	MULTISPECIES: PTS cellobiose transporter subunit IIB	0.443	0.038716903	yes	down
pA_gene0020	virD4	type IV secretory system conjugative DNA transfer family protein	0.428	0.001252881	yes	down
gene2081	prdX	MULTISPECIES: prolyl-tRNA editing protein	0.392	0.001867017	yes	down
gene0382	tyrDC	tyrosine decarboxylase	0.365	0.045319655	yes	down

Table S4. Major differential metabolites detected by using HMDB (Human metabolome database) and Metlin database database. Fold change (FC) represents ratio of level in treatment to control. P-values were calculated by univariate analysis (t-test).

ID	Name	Formula	FC	*P*-value
metab_4591	2-Methylbutylamine	C5H13N	1.081162325	0.003392
metab_7043	PA(18:3(6Z,9Z,12Z)/20:1(11Z))	C41H73O8P	1.06644898	0.0156
metab_4311	CDP-glycerol	C12H21N3O13P2	1.066379479	0.004479
metab_9427	Phosphoenol pyruvate	C3H5O6P	1.066374589	0.04431
metab_1096	Prolyl-Glutamate	C10H16N2O5	1.052436767	0.001585
metab_667	N(6)-Methyllysine	C7H16N2O2	1.046280992	0.01962
metab_4590	Spermidine	C7H19N3	1.045082747	0.005549
metab_161	KAPA	C9H17NO3	1.040786059	0.01807
metab_9435	Aspartyl-Gamma-glutamate	C9H15N3O6	1.033834586	0.02942
metab_9199	Deoxyuridine monophosphate (dUMP)	C9H13N2O8P	1.032010483	0.003677
metab_4271	D-2-Aminobutyric acid	C4H9NO2	1.030345912	0.01283
metab_331	2-Pyrrolidinone	C4H7NO	1.02886057	0.006485
metab_704	D-Tagatose 1-phosphate	C6H13O9P	1.028272444	0.03621
metab_994	7-Methylinosine	C11H15N4O5+	1.027454826	0.02288
metab_2953	4,6-Decadiyn-1-ol	C10H14O	1.027063599	0.01922
metab_1020	Aceglutamide	C7H12N2O4	1.02679803	0.03717
metab_9285	Cis-4-Decenoic acid	C15H24N5O17P3	1.02221824	0.0005556
metab_5685	Uridine 5'-diphosphoglucuronic acid	C15H22N2O18P2	1.022067901	0.03775
metab_3041	2-amino-14,16-dimethyloctadecan-3-ol	C20H43NO	1.021474878	0.01258
metab_4297	Sucrose 6'-phosphate	C12H23O14P	1.020306443	0.00249
metab_5814	Nicotinamide Adenine Dinucleotide Phosphate (NADP+)	C21H28N7O17P3	1.019970292	0.002391
metab_9431	8-(2-{[(2E)-3-(2,4-dihydroxyphenyl)prop-2-enoyl]oxy}propan-2-yl)-2-oxo-2H,8H,9H-furo[2,3-h]chromen-9-yl 3-methylbut-2-enoate	C28H26O9	1.019553528	0.04151

metab_4366	Trehalose 6-phosphate	C12H23O14P	1.019439495	0.01295
metab_2964	25-Acetylvulgaroside	C27H42O7	1.019244604	0.04349
metab_3047	2-Undecen-1-ol	C11H22O	1.018905806	0.02216
metab_1288	3-methyl pyruvic acid	C4H6O3	1.018607328	0.0001358
metab_4360	Betaine	C5H11NO2	1.017837641	0.01225
metab_9446	Glucosylgalactosyl hydroxylysine	C18H34N2O13	1.016672338	0.004303
metab_511	Bis(2-ethylhexyl) phthalate	C24H38O4	1.015488338	0.00328
metab_3918	Phenylacetaldehyde	C8H8O	1.012877747	6.93E-05
metab_3179	Aucubin	C15H22O9	1.012374323	0.00639
metab_9443	5,7-dihydroxy-2-phenyl-8-[3,4,5-trihydroxy-6-(hydroxymethyl)oxan-2-yl]-6-(3,4,5-trihydroxyoxan-2-yl)-4H-chromen-4-one	C26H28O13	1.011944647	0.01689
metab_3068	3-O-Acetylepisamarcandin	C26H34O6	1.010884562	0.004813
metab_788	Uracil	C4H4N2O2	0.9883601	0.02683
metab_9504	Erythronic acid	C4H8O5	0.987798678	0.03408
metab_6336	N-Formylmethionine	C6H11NO3S	0.987374168	0.006624
metab_9132	Hypoxanthine	C5H4N4O	0.987319702	0.01142
metab_4152	2,6-Piperidinedicarboxylic acid	C7H11NO4	0.98680323	0.002638
metab_6057	DTMP	C10H15N2O8P	0.986630636	0.01177
metab_8449	(2R)-2-acetamido-3-(1H-indol-3-yl)propanoic acid	C13H14N2O3	0.985618408	0.008387
metab_1443	Porphobilinogen	C10H14N2O4	0.98506251	0.009729
metab_8661	Hydroxyprolyl-Proline	C10H16N2O4	0.98504806	0.01595
metab_4010	(2S,5S)-trans-Carboxymethylproline	C7H11NO4	0.98488665	0.04198
metab_9218	Olopatadine n-oxide	C21H23NO4	0.98488665	0.0484
metab_8952	Gamma-Glutamylglutamic acid	C10H16N2O7	0.984401709	0.02002
metab_9004	(2S)-2-amino-3-(4-hydroxyphenyl)propanoic acidL-Tyrosine	C9H11NO3	0.984360279	0.002099
metab_5823	Guanine	C5H5N5O	0.984240212	0.01654

metab_286	THTC	C5H8O2S	0.98283859	0.04819
metab_986	L-Homocitrulline	C7H15N3O3	0.981933691	0.03425
metab_8702	Threoninyl-Hydroxyproline	C9H16N2O5	0.980879541	0.03135
metab_1315	Valyl-Tyrosine	C14H20N2O4	0.980651251	0.002793
metab_5124	1-Pyrroline-5-carboxylic acid	C5H7NO2	0.980215538	0.004699
metab_8618	Ketopelenolide a	C15H22O3	0.980183212	0.01044
metab_5250	13-Hydroxy-9-methoxy-10-oxo-11-octadecenoic acid	C19H34O5	0.980133715	0.001198
metab_8933	Glycylprolylhydroxyproline	C12H19N3O5	0.980110724	0.04806
metab_4331	Cytosine	C4H5N3O	0.979749851	0.007449
metab_8636	(2S)-2-amino-3-(1H-indol-3-yl)propanoic acidL-Tryptophan	C11H12N2O2	0.979360357	0.04086
metab_1546	Gamma-Glu-Leu	C11H20N2O5	0.978647687	0.01164
metab_1369	6-Dimethylaminopurine	C7H9N5	0.978568367	0.009298
metab_6715	(+/-)12,13-DiHOME	C18H34O4	0.978243352	0.03827
metab_8810	Xanthosine	C10H12N4O6	0.978139013	0.001221
metab_9579	Gluconic acid	C6H12O7	0.978020106	0.001993
metab_5110	2-Keto-3-deoxy-D-gluconic acid	C6H10O6	0.977839335	0.03068
metab_8926	2-Hydroxy-6-(8,11,14-pentadecatrienyl)benzoic acid	C22H30O3	0.976758045	0.002033
metab_294	Zierin	C14H17NO7	0.976732508	0.001452
metab_5050	Deoxyadenosine monophosphate	C10H14N5O6P	0.976656517	0.007838
metab_1214	Citrulline	C6H13N3O3	0.976588081	0.03397
metab_3354	CL(8:0/8:0/8:0/16:0)	C49H94O17P2	0.975649351	0.03844
metab_3755	2-Pyrrolidone-5-carboxylic acid, methyl ester	C6H9NO3	0.974591329	0.0372
metab_6773	10-Oxooctadecanoic acid	C18H34O3	0.974195893	0.003778
metab_5038	Citric acid	C6H8O7	0.973760132	0.004043
metab_421	Maduramicin	C47H80O17	0.972613582	0.04966
metab_8938	N-lactoyl-Leucine	C9H17NO4	0.972426471	0.003972

metab_3544	Hydroxypentobarbital	C11H18N2O4	0.972385534	0.04318
metab_6408	Gamma-Glutamylphenylalanine	C14H18N2O5	0.97233777	0.003595
metab_8557	3-methyl-2-[(5-oxopyrrolidine-2-carbonyl)amino]pentanoic acid	C11H18N2O4	0.972331369	0.02706
metab_8373	LysoPC(20:5(5Z,8Z,11Z,14Z,17Z))	C28H48NO7P	0.971529057	0.00589
metab_8743	(2S)-2-amino-3-phenylpropanoic acidL-Phenylalanine	C9H11NO2	0.971176018	0.01373
metab_5116	Coenzyme A	C21H36N7O16P3S	0.970450152	0.03535
metab_4867	Adenylosuccinate	C14H18N5O11P	0.970311486	0.02563
metab_8162	9(S)-HODE	C18H32O3	0.969510988	0.004932
metab_4789	Indole-3-carboxaldehyde	C9H7NO	0.969160242	0.00264
metab_332	5-Hydroxy-L-tryptophan	C11H12N2O3	0.968367539	0.000585
metab_8579	Gamma-Glutamylvaline	C10H18N2O5	0.96712374	0.03661
metab_9580	Alanyl-Serine	C6H12N2O4	0.967113276	0.006691
metab_8738	N-lactoyl-Phenylalanine	C12H15NO4	0.96697731	0.01617
metab_3324	Simonin IV	C68H120O24	0.966774602	0.005361
metab_5251	9,12,13-TriHOME	C18H34O5	0.965272979	0.003446
metab_6648	Corchorifatty acid F	C18H32O5	0.964799115	0.02239
metab_1712	Agavoside A	C33H52O9	0.964699157	0.004976
metab_1466	3-[[(2R)-2,4-dihydroxy-3,3-dimethylbutanoyl]amino]propanoic acidPantothenic acid	C9H17NO5	0.964588942	0.04878
metab_391	Lucidenic acid E2	C29H40O8	0.963423555	0.03101
metab_3940	B-D-Glucopyranosiduronic acid	C15H21NO8	0.963222417	0.02069
metab_3380	Permetin A	C54H92N12O12	0.96261543	0.004898
metab_1384	Dopaquinone	C9H9NO4	0.962360507	0.004884
metab_8351	LysoPC(20:4(8Z,11Z,14Z,17Z))	C28H50NO7P	0.960817147	0.003488
metab_8121	Ascorbyl palmitate	C22H38O7	0.96074301	0.0002842
metab_8353	25-Hydroxyvitamin D3-26,23-lactone	C27H40O4	0.959497908	0.002082
metab_1841	Isobutyrylcarnitine	C11H22NO4+	0.95827514	0.008004

metab_389	Lucidenic acid K	C27H36O7	0.956093023	0.02041
metab_5252	DG(8:0/8:0/0:0)	C19H36O5	0.955667594	0.002357
metab_8154	10,11-dihydro-20-trihydroxy-leukotriene B4	C20H34O7	0.95505171	0.0008953
metab_5955	Indole-3-acetic-acid-O-glucuronide	C16H17NO8	0.948259281	0.009738
metab_9040	Xanthine	C5H4N4O2	0.94750105	0.01182
metab_8854	Niazicinin A	C17H23NO8	0.94722702	0.01099
metab_5962	Ginkgolide J	C20H24O10	0.946842441	0.007341
metab_3465	2-Pyridylacetic acid	C7H7NO2	0.944850498	0.02589
metab_1579	L-cis-Cyclo(aspartylphenylalanyl)	C13H14N2O4	0.942373564	0.04856
metab_8752	Dephospho-CoA	C21H35N7O13P2S	0.940161968	0.0002302
metab_9284	2-Ketoglutaric acid	C5H6O5	0.939099385	0.01851
metab_8307	Leukotriene C5	C30H45N3O9S	0.9342822	0.0003483
metab_1458	D-Pantetheine 4'-phosphate	C11H23N2O7PS	0.934068059	4.66E-06
metab_3670	Succinoadenosine	C14H17N5O8	0.931478405	0.005683
metab_5885	Gamma-Glu-Cys	C8H14N2O5S	0.900813331	6.21E-07
metab_10032	Glutathione	C10H17N3O6S	0.884861407	0.01429
